# Clinical utility of cardiac magnetic resonance T2 mapping for acute myocardial edema

**DOI:** 10.1186/1532-429X-13-S1-O101

**Published:** 2011-02-02

**Authors:** Asad A Usman, Marie Wasielewski, Jeremy D Collins, Mauricio S Galizia, Andrada R Popescu, James C Carr

**Affiliations:** 1 Northwestern University, Chicago, IL, USA

## Objective

To evaluate the potential clinical utility of T2 quantitative mapping for myocardial edema in acute disease pathology - myocarditis, myocardial infarction, Tako-Tsubo cardiomyopathy, and transplant rejection.

## Background

Edema is a generic tissue response to acute myocardial injury and, therefore; a potential marker of impending tissue damage. Currently in clinical use, T2 weighted imaging provides a qualitative technique in assessing myocardial edema. We hypothesize that quantitative T2 mapping in patients with suspected cases of myocarditis, myocardial infarction (AMI), and cardiac transplant rejection will provide a more sensitive and specific diagnostic prediction than with T2W imaging, and add to other imaging techniques.

## Method

We used a non-contrast multiplanar single-shot and cine TrueFISP and steady state free precession (SSFP) sequence developed by Siemens Healthcare which consists of T2-prepared, linear flip angle series of 20, 60, and 90 degrees, and SSFP readout. ECG triggered breathold T2 mapping was done on the three short axis and 4 chamber images on a 1.5 T scanner (MAGNETOM Avanto, Siemens Erlangen, Germany). Regions of interest (ROI) based on the 17 segment myocardial model were drawn by two blinded reviewers (AU and MW) for inter-rater and intra-rater reliability.

## Results

**Figure 1:**
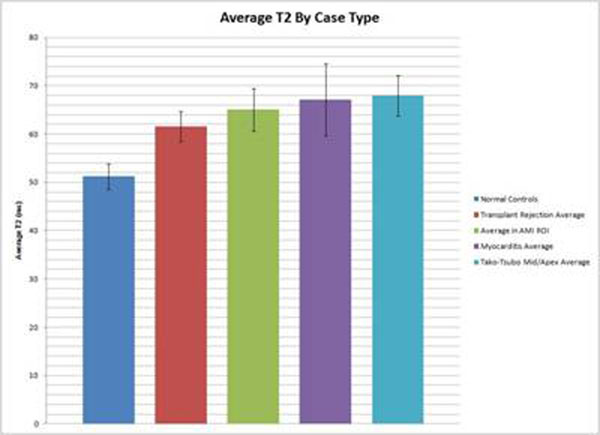


Complete results are presented in table 1. T2 mapping was done on 6 suspected acute myocarditis, 13 AMI, 1 Tako-Tsubo cardiomyopathy, and 25 transplant patients. The average age of all patients was 50.9 ± 7.8 years old, 62% male, and average scan time was 45 minutes. Control studies on normal patients demonstrated a global T2 mapping average of 51.2 ± 2.7 ms. Of the 6 patients with suspected myocarditis 3 were found to have no myocarditis based on MR criteria and clinical follow-up (100% concordance with T2 mapping). The 3 patients with myocarditis had cardiac sarcoidosis, viral, and giant cell myocarditis with average T2 of 70.6 ± 6.7 ms (p<0.05). 10/12 suspected AMI patients had segmentally concordant T2 values in relation to delayed enhancement outcomes. The average infract segment T2 value was 66.4 ± 3.2 ms (p<0.05). The basal T2 value in the Tako-Tsubo case was 52.3 ± 3.2 ms, while the mid and apical average were 68.0 ± 4.2 ms (p<0.05). Figure 1

**Table 1:** Demographics

Total cases	65				
Group	Controls	Transplant	AMI	Myocarditis	Cardiomyopathy

Average age	32.7 ± 7.3	55.8 ± 13.8	55.5 ± 10.3	49.2 ± 13.2	60
Percent female	50%	44%	33%	33%	100%
Normal**	20	22	4	3	0
Disease	NA	3	8	3	1
Total	20	25	12	6	1
Outcomes					
Average T2 Normal ROI (ms)	51.2 ± 2.7	52.1 ± 2.4	54.1 ± 3.1	54.0 ± 3.7	52.3 ± 3.1
Average T2 Disease ROI (ms)	NA	61.6 ± 3.1	65.0 ± 4.4	67.1 ± 7.5	68.0 ± 4.2
Significance	NA	<0.05	<0.05	<0.05	<0.05*
Ejection fraction	62%	58.7%	39.$%	41/1%	32.2%

*p-value compared internally from basal segment to mid and apical segment

** Normal represents cases that were suspected to have outcome, i.e. AMI, myocarditis, etc, but were by clinical assessment MRI, and cellular pathology determined not to have any disease. These cases T2 Mapping were compared to cases with in-group pathology.

## Conclusions

T2 quantification provides a useful clinical tool in evaluating cardiac pathology with acute cellular responses and edema. We hope with advances in spatial and temporal resolution T2 mapping can be used to determine myocardial viability in cases of AMI, predict transplant rejection, or help rule-out myocarditis.

